# DockNmine, a Web Portal to Assemble and Analyse Virtual and Experimental Interaction Data

**DOI:** 10.3390/ijms20205062

**Published:** 2019-10-12

**Authors:** Ennys Gheyouche, Romain Launay, Jean Lethiec, Antoine Labeeuw, Caroline Roze, Alan Amossé, Stéphane Téletchéa

**Affiliations:** UFIP, Université de Nantes, UMR CNRS 6286, 2 rue de la Houssinière, 44322 Nantes, France; ennys.gheyouche@univ-nantes.fr (E.G.); romain.launay1@etu.univ-nantes.fr (R.L.); jean.lethiec@gmail.com (J.L.); antoine.labeeuw@etu.univ-nantes.fr (A.L.); caroline@affilogic.com (C.R.); alan.amosse@gmail.com (A.A.)

**Keywords:** protein–ligand analysis, drug discovery and design, structure–activity relationships

## Abstract

Scientists have to perform multiple experiments producing qualitative and quantitative data to determine if a compound is able to bind to a given target. Due to the large diversity of the potential ligand chemical space, the possibility of experimentally exploring a lot of compounds on a target rapidly becomes out of reach. Scientists therefore need to use virtual screening methods to determine the putative binding mode of ligands on a protein and then post-process the raw docking experiments with a dedicated scoring function in relation with experimental data. Two of the major difficulties for comparing docking predictions with experiments mostly come from the lack of transferability of experimental data and the lack of standardisation in molecule names. Although large portals like PubChem or ChEMBL are available for general purpose, there is no service allowing a formal expert annotation of both experimental data and docking studies. To address these issues, researchers build their own collection of data in flat files, often in spreadsheets, with limited possibilities of extensive annotations or standardisation of ligand descriptions allowing cross-database retrieval. We have conceived the dockNmine platform to provide a service allowing an expert and authenticated annotation of ligands and targets. First, this portal allows a scientist to incorporate controlled information in the database using reference identifiers for the protein (Uniprot ID) and the ligand (SMILES description), the data and the publication associated to it. Second, it allows the incorporation of docking experiments using forms that automatically parse useful parameters and results. Last, the web interface provides a lot of pre-computed outputs to assess the degree of correlations between docking experiments and experimental data.

## 1. Introduction

There is a booming demand to develop precision medicine products, that is, to design new drugs targeting regular or pathological protein variants [1,2,3]. These targeted strategies are very promising for the treatment of cancers and other diseases but are very challenging to set up [4]. First, it is necessary to assemble the knowledge on biological processes of interest, in order to identify which protein should be specifically targeted by new drugs [5]. Second, a review of known ligands, be them agonists or antagonists, is essential to identify key binding motifs [6]. Last, when possible, one needs to gain as much as possible insight into the protein three-dimensional structure obtained by crystallography or NMR and the allostery associated with the protein [7].

It takes time and expertise to get a broad overview of the protein to target and of its specific modifications related to a disease. The limitations in this process comes from the immense gap of knowledge that individuals in one laboratory can apprehend, in comparison with the ocean of data available in scientific literature. Fortunately, in order to link the experimental activities of various small chemical entities with their (protein) targets into databases, there is a strong ongoing effort to organise these data logically by human experts, a process called curation. Once set up, these databases can be queried via their web interface but also queried using dedicated programmatic access for batch data retrieval [8,9].

An important limitation for gathering experimental results for a target comes from the standardisation of experimental data, of target names and of small chemical entities. For example, it is common practice to reference a molecule by a common name in a given laboratory, to use a chemical name or to name it based on the biological process interrupted by the drug. These difference in protein nomenclature is visible for the Tartget Of Rapamycin (mTOR) where the protein itself is referenced as mTOR [10] but the protein name in Uniprot is Serine/threonine-protein kinase mTOR. This variety of definitions for small chemical entities and protein targets is not important when people are working closely together but this renders the comparison of data very difficult between laboratories.

Before being publicly available, either published or patented, compounds synthesized or in tests have to stay private. In the meantime, the teams of biologists, chemists and chemoinformaticians/structural biochemists need to collaborate to bring together their results in a comprehensive and efficient way. This requirement of privacy and collaborative methodology starts to be critical when the collaborators are split in different and sometimes geographically dispersed teams.

We have set up the dockNmine portal (http://www.ufip.univ-nantes.fr/tools/docknmine/) to ease the data management, exchange and analysis of project-based medicinal chemistry studies. The portal allows to manage private experimental data and private docking studies but also makes use of public data when possible for homogenising proteins description and small molecules activities. We now describe dockNmine organisation and implementation.

## 2. Results

**Figure 1:**
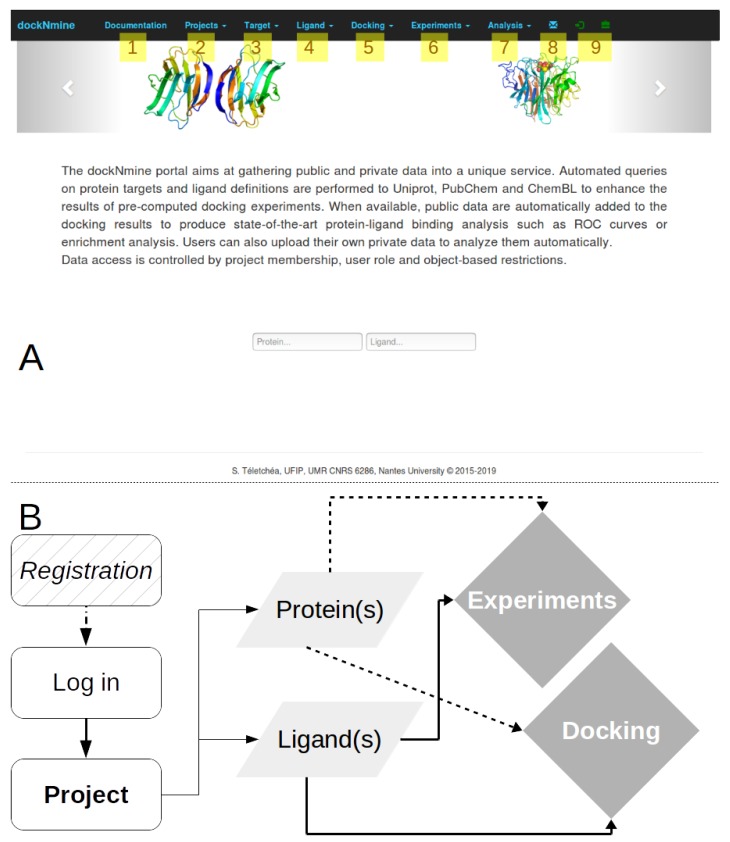
Overview of dockNmine. (**A**) (1) Link to the documentation of each service; (2–7) access to each service independently; (8) contact link; (9) login and registration links. A simple demonstration of the functionalities is accessible upon connection using the demo account using the log-in glyphicon (login: *demo*, password: *demo*) or by registering upon clicking on the briefcase; (**B**) Once connected, the user can create or join a project where all his data will be assembled (rounded-corner rectangle), he shall then add required protein and ligands (parallelogram) and link them to experimental data and docking results (diamond).

The dockNmine home page is divided into six independent services to ease a logical workflow for processing docking and experimental data (Figure 1A). After a broad overview of dockNmine organisation, a detailed explanation of its services is provided in dedicated sections.

### 2.1. DockNmine Overview

The portal philosophy is directly inspired from funded-based projects, therefore it is designed to isolate independent and confidential data from different users. This management by project allows to assemble ligands, protein(s), docking and experimental data into a coherent ensemble, via dedicated feature-control checks. Once registered and connected, the user can start a new project or join an existing one. In both cases, either a private project or a shared project within a small group, the connected used can start to organise his computational data using dockNmine services. We have set up as an example a new project called “Target” created by the user “demo” by following the workflow presented in Figure 1B.

**Figure 2:**
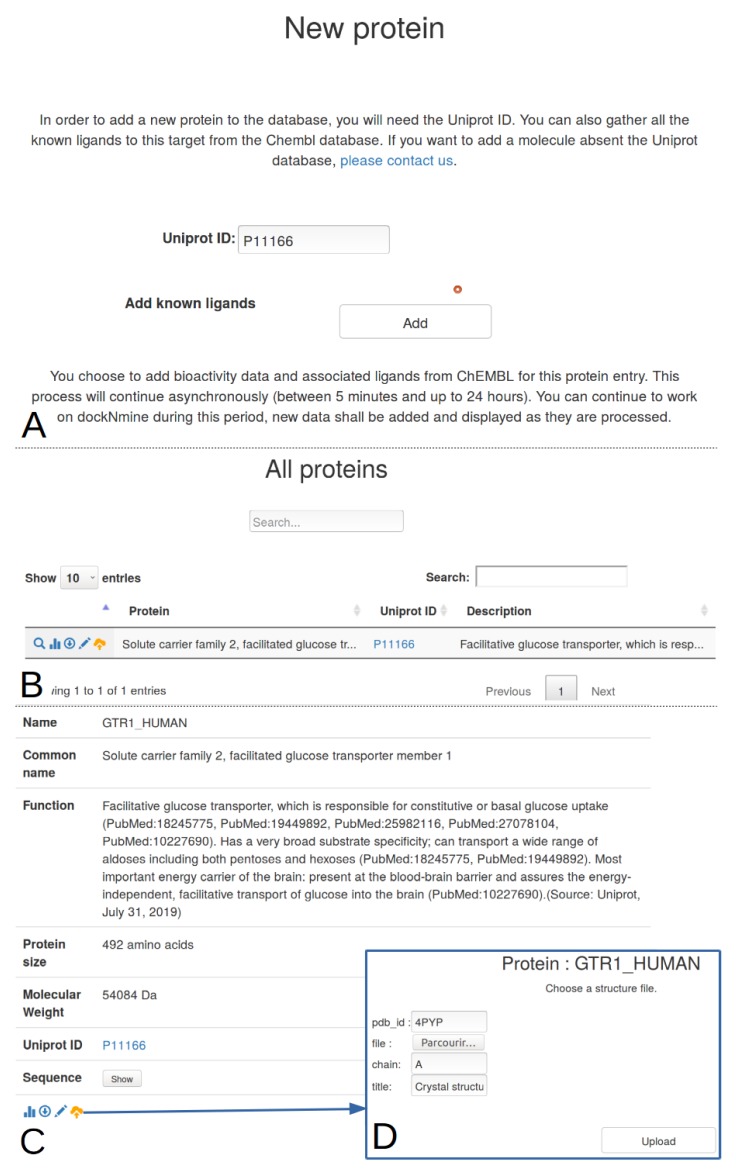
Automated target management in dockNmine for Solute carrier family 2, facilitated glucose transporter member 1. (**A**) The screenshot presents a request for the retrieval of data for the Uniprot ID P11166 and of known ligands from ChEMBL; (**B**) A condensed view of the targets for the project is provided. Some glyphicons are provided to see the details of the entry (magnifier icon), to get detailed statistics (histogram), to download existing data in csv format (circled arrow), to add a comment (pen) or to add a three-dimensional structure for the target (orange arrow towards a cloud); (**C**) Detail of a given entry; (**D**) If required, the user can upload one or many structures for the target. As structure files can be processed in virtual screening experiments, only the structure file is mandatory, all other fields being optional.

**Figure 3:**
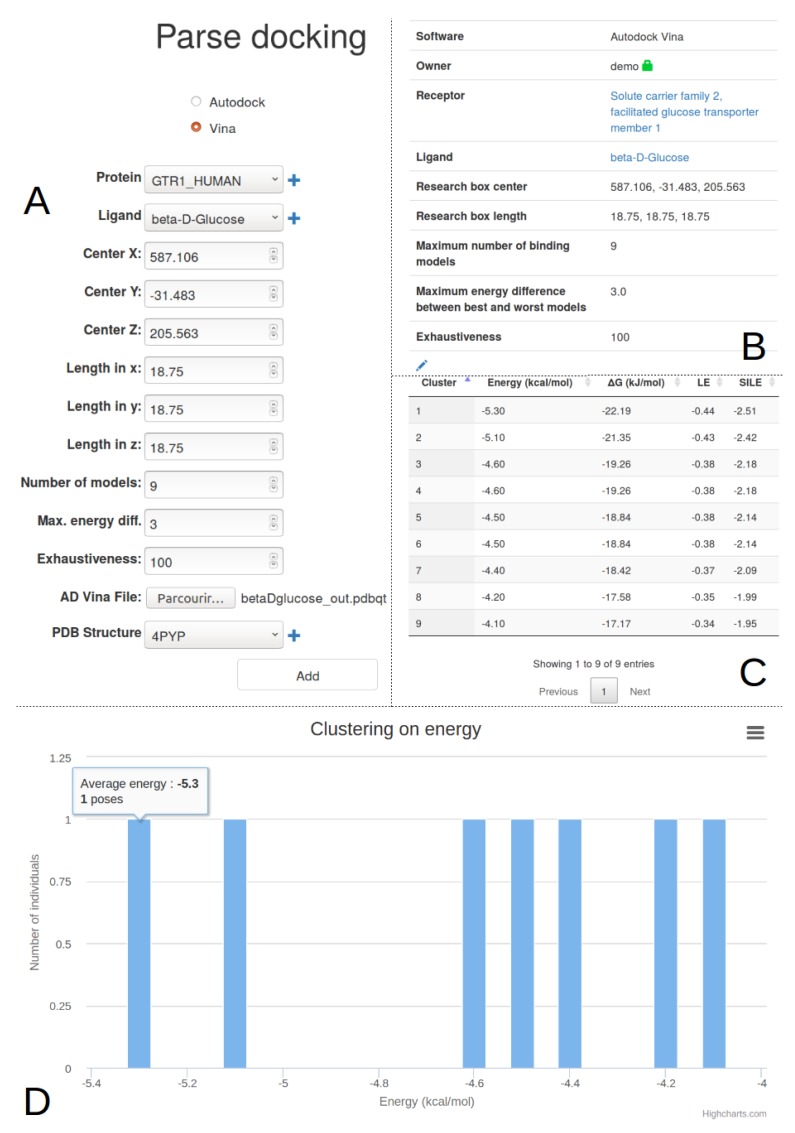
Vina docking results import for the beta-D-glucose docked in the glucose transporter. (**A**) Upon selection of the docking method, a dedicated form allows to link protein, ligand and docking results. Detailed docking parameters must be provided to allow a further comparison of docking profiles between experiments. If required, the plus glyphicon allows to add a target, a ligand or a target structure prior to entering the docking results; (**B**) Detail of the docking analysis. This view indicates the principal features of the docking method; (**C**) After docking processing, the cluster energy of vina is transformed into kJ/mol, LE and SILE automatically, to ease comparison against other experiments or other ligands; (**D**) Interactive graph depicting the discrete cluster and associated energy. This graph, which can be easily downloaded as an image, allows a rapid overview of the docking energy dispersion for the ligand.

The user can now use the Target tab (Figure 1A3) to add the description of its target from a unique identifier, for example the Solute carrier family 2, facilitated glucose transporter member 1, whose uniprot ID is P11166. A request is triggered on uniprot to retrieve its protein name, its description and other parameters (Figure 2B). This protein is automatically added to the main project as its first target. This transporter allows the exchange of glucose and is important for glucose supply in brain and other organs [11,12,13]. The beta-D-glycopyranose is referenced under the ID 64689 in PubChem, this ligand is added via the form provided under the Ligand tab (Figure 1A4) using the “Add a ligand” link. The dockNmine request triggers a query on PubChem to download reference ligand information and the file containing its three-dimensional structure in sdf format. This file is processed using rdkit to generate additional descriptors. The summary page for known ligands shows the result of this process (Figure 1D). Since the crystal structure of GLUT1 contains another ligand (B-nonylglucoside), this ligand was also incorporated into dockNmine. These ligands were screened on the GLUT1 structure (PDB ID: 4PYP) using vina (see Supplementary Materials) and the output of vina is incorporated using the Docking service. To upload a complete docking result, its structure file has to be previously uploaded to the target but this step is more extensively described below (Figure 2D). Once all mandatory parameters are present, the docking information is added to dockNmine (Figure 3).

The validity of a virtual screening must be assessed by comparing its results with experimental data. Some experimental data are already openly aggregated by entities like BindingBD or ChEMBL but this information can be retrieved automatically during the target addition to dockNmine. Though, there is a important amount of data impossible to share immediately, mostly coming from internal experiments in the laboratory. The Experiment service allows to include data from the laboratory and its partners without the need to disclose it too early. We have included into the demo account a piperazin derivative where a cellular IC50 was determined of 50 nM is referenced in Binding DB (ID 50155745) from the work of Siebeneicher and co-workers [14]. The structure file for this compound was incorporated from PubChem using the identifier 1387075.

### 2.2. Target Management

A more extensive explanation of the incorporation process and data management is provided in this section for the addition of a given target. As indicated above, we study the Solute carrier family 2, facilitated glucose transporter member 1 (Uniprot ID P11166). Within a given project, due to the variety of disciplines involved, scientists may refer to a target using an acronym, its gene name or a common name. These names are prone to change so we have provided a simplified way to incorporate a given target from its reference uniprot identifier, as shown in Figure 2. By clicking on Target-> Add a protein, dockNmine retrieves automatically from Uniprot the target name, common name, function, size, molecular weigth and sequence. A checkbox is also provided to grab bioactivities of ChEMBL compounds for the target using ChEMBL web services [15,16] but the addition of ligands is processed asynchronously to allow a better experience in the interface. The process can be sufficient if only experimental data are to be added for a target but if docking results need to be incorporated, then a reference structure file is required. This addition is accessible by clicking on the orange glyphicon (Figure 2B,C). Since the structure file often needs to be extensively processed for docking [6], only the resulting structure file in pdb or cif format is required. We have uploaded for the purpose of demonstration the unprocessed crystal structure of the glucose transporter resolved by Deng and colleagues [11]. Using the target management service, the user is able to incorporate most important data in few minutes.

### 2.3. Ligand Import And Management

There are two ways to manage ligand incorporation into the database—(i) by adding one ligand at a time via the dedicated form, (ii) by uploading multiple ligands from a file, which will be regrouped into a library.

**Figure 4:**
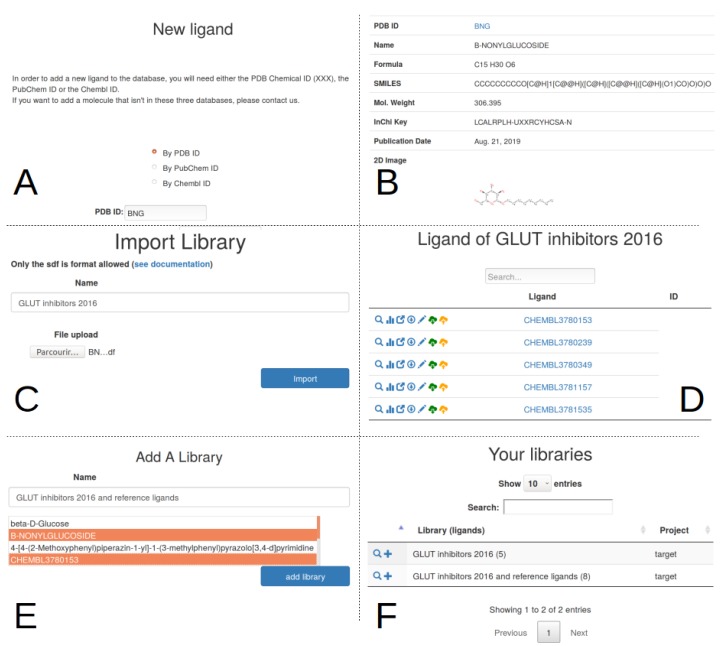
Additions of a single ligand or of multiple ligands into a library. (**A**) The form allows to add a single ligand from either the Protein Data Bank (PDB), PubChem or ChEBML. The query using the PDB request is shown here; (**B**) After a short period, the details of the added ligand can be accessed, if available, a 2D depiction of the molecule is displayed; (**C**) For more extensive data incorporation, the simplest way is to add a library and alongside a valid sdf file; (**D**) In this case all ligands available in the sdf file are processed, de-duplicated and added to the library; (**E**) If necessary, a small subset of ligands can be arranged in another library; (**F**) This new library will be referenced.

The single ligand form (Figure 4A) allows to incorporate ligand information from the Protein Data Bank (PDB) [17], PubChem [8] or ChEMBL [9]. The crystal structure of the glucose transporter contains a glycosyl analogue, b-nonyglucoside, used to trap the protein in an inward-open conformation [11]. This ligand has the identifier BNG in the PDB. When selecting the PDB input format and searching for BNG, a query on the PDB is performed to retrieve the ligand name, chemical formula, SMILES notation, molecular weight and InChiKey. The ideal three-dimensional coordinates of the molecule is downloaded in sdf format and added as the reference conformation for the ligand. By default the visibility of any ligand entered using the single ligand form is restricted to the project members only.

If multiple ligands are to be added rapidly, that is, from a commercial supplier, another possibility is to create a dedicated library from a multiple-compound sdf file (Figure 4C). For example, Siebeneicher and colleagues [14] have determined a series of GLUT inhibitors involving piperazine derivatives. These results are available in ChEMBL in the document report card CHEMBL3779893. Five compounds were downloaded in sdf format from the ChEMBL report (Table 1) and assembled into a single sdf file. This library was incorporated with a free text name using a file upload form (Figure 4C), resulting into the addition of the five new ligands into the database, joined into the “GLUT inhibitors 2016” library. To stress the system, the incorporation of a larger library of >11,000 compounds was assessed (data not shown). In this situation, the rate for ligand processing was about 100 ligands/s. The library facility can also be used to regroup existing individual ligands into a coherent ensemble (Figure 4E,F). This approach allows to delineate sub ensemble of ligands for easier data extraction and analysis but has no strong dependence on the classified ligands. It is therefore easy to remove a library, this will not cascade to the removal of ligands or of ligands data.

In both ligand import processes, it is important to not duplicate ligands even if different names are found, coming from the diversity of upstream sources. Instead of relying on ligand names, we have chosen a more robust approach by comparing the InChiKey [18] of new ligands to existing ones. If the InChiKey is not available, a Morgan fingerprint [19] is computed using rkdit [20] and used for comparison.

### 2.4. Docking Import And Management

The docking process needs a large amount of computational power to screen libraries of ligands against a given target. We envision to provide the possibility of performing some dockings through the interface of dockNmine in future revisions for a limited set of molecules but the potential ressources required are not yet available. Up to now, it is however possible to record already existing vina or autodock [21,22,23] results. This process already allows to standardize ligand import and management and to perform basic analysis. The course of action for pre-computed docking data is detailed hereafter.

The user needs to define the target, the ligand and the structure file used for docking. Depending on the docking software, autodock or vina, different information needs to be provided. The completed input form is presented in the Figure 3A. It is possible to not enter all docking parameters but it is recommended to add them all in order to be able to compare different docking experiments for the same partners (target+structure and ligand). Upon form submission, the pdbqt vina output file is parsed to extract the cluster number and its associated energy for each pose. Once processed, the user is redirected to the docking list page where he can inspect the incorporated docking by clicking on the magnifier icon.

This magnifier icon redirects to the details of the processed data (Figure 3B–D) where not only the docking parameters are listed but also the extracted energy by cluster (and/or pose if autodock vina is used). The cluster energy is transformed into kJ to allow a rapid comparison with other experiments and two indicators are also computed—Ligand Efficiency (LE) and Size-Independent Ligand Efficiency (SILE) [24]. These indicators were developed to compensate the tendency of large ligands to obtain better docking scores based on the ligand size rather than being effectively more active experimentally. These two measures are nowadays discussed or further explored [25] but we have chosen to not provide additional indicators in the table since more advanced features are available under the analysis tab.

This single-step ligand incorporation mechanism is perfectly fit to compare ligands or docking parameters for a small amount of compounds. Within minutes, it is possible to arrange properly and formally the docking data without expert needs. After the output ligand file is uploaded in pdbqt format, all other steps are automated to ease the user experience.

### 2.5. Experimental Data

**Figure 5:**
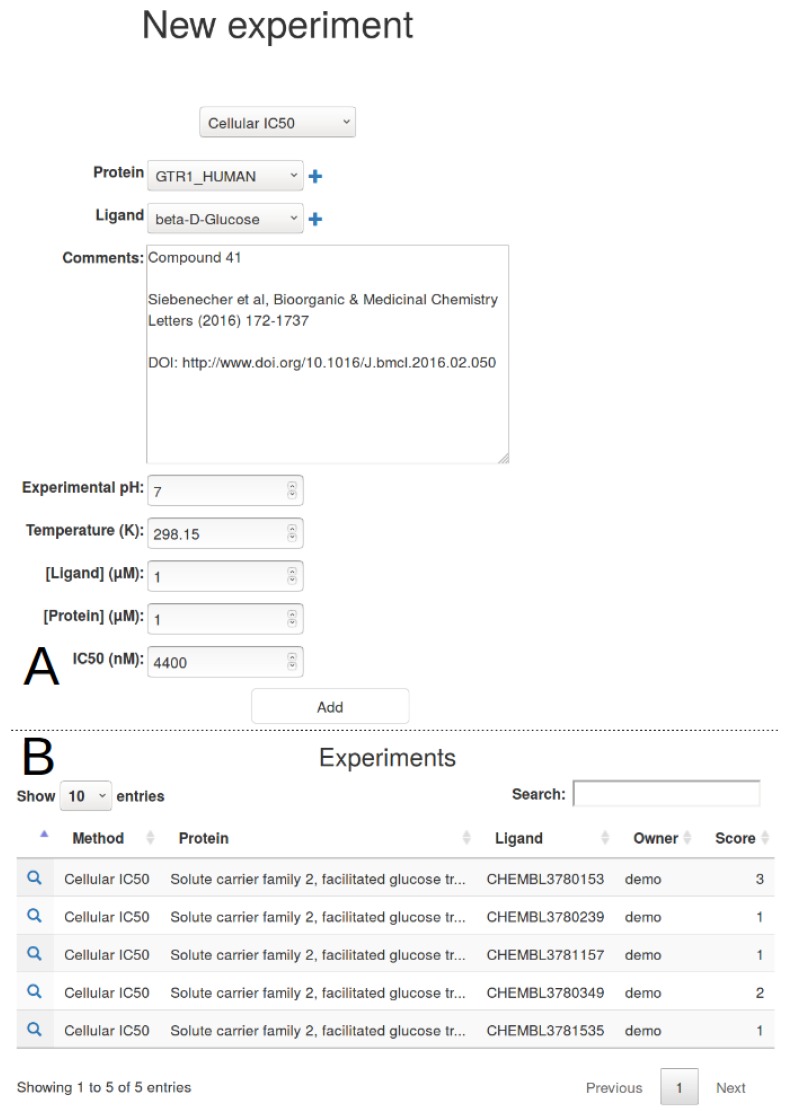
Experimental data addition for a selection of ligands from the study of Seibeneicher and co-workers [14]. (**A**) After IC50 selection from the drop-down menu, a method specific form is shown to the user. Pre-defined valued are provided for pH, temperature, target and ligand concentrations since they are seldomly used. The user can complete the free text box to indicate the data origin, either being from literature of from private laboratory experiments; (**B**) Upon form validation, all experimental data are listed, with an auto-computed normalised score important for comparison with virtual predictions.

Although virtual screening may be useful for finding the needle in a haystack, it is important to rely on experimental validation to assess the predictive power of calculations. The experimental tab allows to add to individual ligand experimental results for six different experiments—(i) IC50; (ii) hemaglutination; (iii) isothermal titration calorimetry (ITC); (iv) surface plasmon resonance (SPR); (v) fluorescence anisotropy; (vi) affinity chromatography (Figure 5). This initial list can be easily extended to adjust to a specific method but should already be generic enough to register most of experimental data. A large comment box allows to indicate the method in detail and/or the reference study. This free-text addition is important to keep track of a given laboratory result prior to publication, altogether with better procedures for chemical names and entities across partners of the project.

### 2.6. Library Analysis

**Figure 6:**
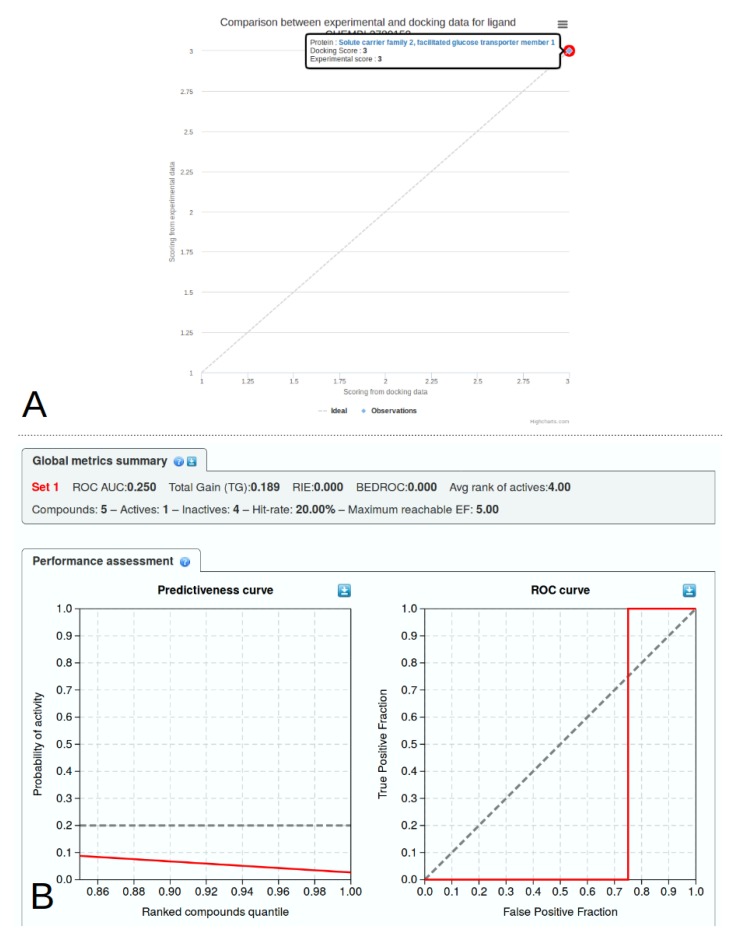
Analysis of ligand classification using reference methods. Experimental data were taken from the work of Seibeneicher and co-workers [14], the docking results were computed for this study. (**A**) Single ligand analysis for CHEMBL3780153. Both the experimental and docking values allow to classify it as a good ligand; (**B**) A more complete analysis of the overall virtual screening allows to evaluate the ongoing project evolution.

Virtual screening studies theoretically provide ligand-binding predictions in close agreement with known experimental data. It is however difficult to compare virtual results directly with experimental values. First, the free energy of binding is often tuned and estimated from a limited set of ligands (autodock, vina), which may be largely unrelated to the study of interest. Second, not a lot of study provide direct measure of ligand-protein interaction with a defined kD or ki. Third, even if the binding of a chemical entity is a direct measure, for instance using surface plasmon resonance (SPR) or thermocalorimetry (ITC) methods, the binding consequence can lead to different definitions, the ligand being classified at least as an agonist or antagonist, not counting the partial or reverse definitions and receptor allostery [26]. We have incorporated interactive indicators to provide a better insight into the predictive power of docking experiments (Figure 6). The Receiver Operating Characteristic analysis (ROC) [27] is useful in rapid evaluation of the docking performance by comparing True and False prediction rates. ROC curves are provided per ligand (Figure 6A) or synthetically for a given target in the detailed target page. For a more expert analysis of docking performance against experimental results, advanced features are displayed under the analysis menu. These analysis based on clinical epidemiology were defined by Empereur-Mot and his colleagues [28] and a demo is provided online (http://stats.drugdesign.fr/). For both analysis, it is important to rank the ligand according to the virtual and experimental results. We have defined three classes for ranking ligands—(i) good; (ii) intermediate; and (iii) bad. For docking results, the good category is reported if the autodock or vina free energy (kcal/mol) of the best cluster is ≤−10, an intermediate ligand lies in the interval −10 > energy ≤−6.5 and a bad ligand has a lower free energy of binding. The corresponding thresholds for a IC50 (nM) result are ≤100 for a good ligand, >100 and ≤1000 for an intermediate ligand, and above 1000 for a bad ligand. Pre-defined thresholds are also provided for each experimental result which can be incorporated in dockNmine. The experimental and virtual categories are then compared to indicate if there is an agreement between predictions and results. This information is then transformed automatically for being displayed in the dedicated graphs.

### 2.7. Access Controls

Any user can freely discover dockNmine without authentication but a demonstration account is provided to evaluate all of its functionalities. Once connected or registered using the log-in or the briefcase glyphicons (Figure 1A), the user is attributed a **Project Manager** role (Table 2) and can therefore create a new project, mandatory to start adding data in dockNmine. Once set up, the Project Manager can share the project credentials with collaborators to allow them to join the project. The access controls systems is a combination of Guardian rules and of permissions offered by Django’s internal group management system. A lot of predefined permissions can thus be finely tuned to Create, Read, Update and Delete data (CRUD) on any object or data in the database. Since this granularity may be hard to apprehend, we have set up pre-defined roles (Table 2) where these permissions are clearly split into viewers (Project Member) and editors roles (Project Manager) with a clear separation of privileges. To ease dockNmine usage, by default all new accounts have a Manager role but it is recommended to restrict this default role to **Member** for some collaborators in order to avoid errors. Only the principal dockNmine administrator has full control over the service and can adjust permissions for users, objects and data.

Before adding any information, connected users have to select an existing project. This mechanism allows to automatically define data visibility and origin, as all members of the project can see targets, ligands, dockings, experiments and their automated analysis. This per-project access allows to restrict data visibility to project members and accross projects. Pre-defined Manager and Member roles are indicated in the login page to demonstrate the difference between them. With a Member role, the user can add a ligand to the library but cannot upload a specific structure file. With a Manager role, the user can upload its own sdf file, carefully prepared using an external software or web service.

### 2.8. Extending Docknmine

This web server is meant to assemble various docking experiments and to compare it with existing or *in-house* experimental data. Since there is a large variety of virtual and experimental methods, it is possible to update dockNmine to take them into account. Some expertise is required in Django development so users are advised to first contact the corresponding author.

## 3. Discussion

One of the biggest challenges when trying to reach the precision medicine objectives, the goal to provide a per-individual efficient treatment, is the need to take into account little variation in protein sequences in order to predict if these mutations will lead to dramatic changes in the protein structure [29]. This research field, also known as structural genomics [30,31], is developing rapidly though many technological obstacles still need to be leveraged to be applied blindly [32]. In order to illustrate how dockNmine could properly be used to integrate results from these approaches, we have detailed its independent services allowing to assemble existing public and private knowledge into logically organised comprehensive data sets.

### 3.1. Single Protein Analysis

We chose the GLUT1 receptor (Solute carrier family 2, facilitated glucose transporter member 1) as an example. We indicated how to add this protein entry into the portal and how to add virtual and publicly available experimental data from databases and scientific literature [9,14]. We provided original docking data (see Supplementary Materials for details) to exemplify how one should make use of the portal. If one was willing to reproduce these steps, much more attention would have to be paid to the virtual screening experiments for getting relevant results [6]. Since these steps require manual expertise and computational time, we have not allowed docking computation to be performed directly within the portal. This situation may change but there are already efficient and popular solutions available for users interested in performing large virtual screening studies [33,34,35,36]. One of the critical steps for setting up a virtual screening approach is related to ligand preparation, classification and ranking. There are many challenges for each of these steps but again a lot of reliable solutions exist [37,38]. In the end, users can also make use of commercial software which provide a lot of facilities for setting up virtual screening studies, by pipe-lining all these steps silently.

### 3.2. Multiple Proteins Analysis

**Figure 7:**
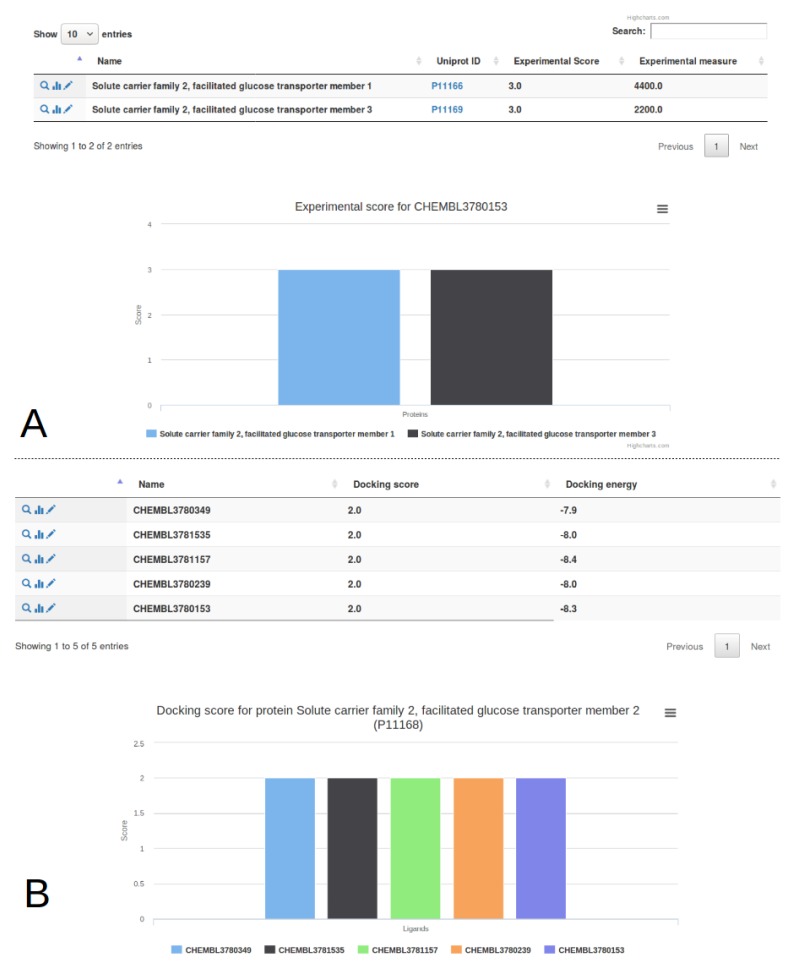
Comparison of experimental and virtual data for GLUT proteins and their ligands. Experimental data from Seibeneicher and co-workers [14], the docking results were computed for this study. (**A**) Comparison of ligand results for CHEMBL3780153. The docking was performed on all proteins but experimental values are only available for GLUT1 and GLUT3; (**B**) Tabular results and graphical representation of docking results for GLUT2.

In order to get a broader overview of the GLUT family response to different molecules, we have processed two other glucose transporters, GLUT2 and GLUT3, for which experimental binding data are also provided in the reference article [14]. The crystallographic structure of GLUT3 was determined by Deng and colleagues (PDB ID: 4ZW9) [39], the model of GLUT2 was downloaded from Swiss-Model (automatically computed from the structure 4ZXC) [40]. We selected a crystallographic structure and a publicly available model to compute docking energies with vina for illustrating how one could compare his experimental data with predictions without advanced expertise on computational protein modelling. The incorporation of these proteins, of virtual screening data and of experimental information were processed as previously described. These results are available by ligand (Figure 7A) or by target (Figure 7B) from the drop-down options of the Analysis tab. These views present individual graph and table for dockings and experiments. For the ligand CHEMBL3780153, the experimental IC50 binding values are 4400 nM for GLUT1 and 2200 nM for GLUT3, no data being available for GLUT2. By selecting the GLUT2 receptor from the menu, the predicted binding value of the best pose is −8.3 kcal/mol, ranking it as a medium binder on the target according to our ranking procedure. Since the docking value allows to rank this ligand in the lowest binding category (rank 3) for both GLUT1 and GLUT3, in agreement with experimental data, this rapid comparison suggests a better recognition of CHEMBL3780153 by GLUT2. This simple comparison of docking and experimental results could be exact but the user is warned about the limited predictive power of the included dataset, with a negative predictiveness and an Area Under the Curve (AUC) under 0.5 for GLUT1 (Figure 6B).

To move towards precision medicine, dockNmine can act as a central gathering portal to add much more experimental and docking information. In the case of the GLUT members, this would required adding docking and experimental data for the 14 members of the SLC2 family, from the recently published study in Reference [41,42].

### 3.3. Advanced Analysis

The provided analysis pages are simple and standard methods for comparing docking and experimental data. Since these views may not be sufficient, we offer to download the complete data set in the detailed target and ligand pages, by clicking on the download glyphicon. In this case, docking and experimental data are arranged into a convenient csv file for further processing in any spreadsheet.

The existing portal already allows to assemble a lot of knowledge seamlessly. As more information may be required for further deciphering protein-ligand interaction, for instance for advanced machine learning processing [43], new features shall be incorporated [44]. Some of future improvements may comprise the addition of direct docking computation from the interface, advanced protein-ligand analysis [45] and visualisation [46] and other functionalities demanded by users.

## 4. Materials and Methods

### 4.1. Server Design, Implementation And Security

The django framework (https://www.djangoproject.com/) was used to arrange data into dedicated classes. Access controls are ensured by django’s built-in system supplemented by the guardian module to provide per-object control. Each page in the interface is submitted to permission validation ensured by a dedicated decorator developed specifically for this purpose. User interaction and interactive displays are provided using Bootstrap (http://getbootstrap.com/) and jQuery (http://jquery.com/). The specific protein-ligand global statistics analysis is derived from the work of Empereur-Mot and collaborators [28].

### 4.2. Data Retrieval And Processing

Queries on external databases are executed using the python3 requests module. When available, public API are used like for ChEMBL for instance [9], otherwise simple HTTP request are performed. Queries are then processed using biopython [47] for proteins and rdkit [20] for small chemical entities.

## 5. Conclusions

With the need to address large-scale, diverse and targeted protein-ligand interaction predictions, it is essential to be able to quickly assemble public and private experimental and virtual data. The dockNmine portal aims at providing the first component for this ambitious goal; it is freely accessible at the http://www.ufip.univ-nantes.fr/tools/docknmine/.

## Figures and Tables

**Table 1:** Selected piperazine derivatives from the work of Siebeneicher and colleagues [14] and their reference in ChEMBL. The vina free energy value is indicated for the best cluster, see Supplementary Materials for calculation details.

Compound ID	IC50 (nM)	ChEMBL ID	PubChem ID	Vina Energy (kcal/mol)
**13**	1	3780239	72547759	−6.2
**3**	25	3781157	1977736	−7.4
**66**	80	3781535	127030174	−5.3
**63**	510	3780349	52149799	−6.3
**41**	44,000	3780153	127030188	−5.3

**Table 2:** CRUD permissions management in dockNmine.

	Project	Target	Ligand	Experimental Method	Docking	Library
SuperUser	CRUD	CRUD	CRUD	CRUD	CRUD	CRUD
Manager	CRU	CRU	CRU	CRU	CRU	CRU
Member	R	R	CR	CR	CR	CR
Anonymous	R	R	R	R	R	R

## Supplementary Materials

The following are available online at https://www.mdpi.com/1422-0067/20/20/5062/s1, Supplemental Methods S1: Receptor and ligand setup.

## Abbreviations

The following abbreviations are used in this manuscript:

AUC	Area Under the Curve
CADD	Computer-Aided Drug Design
CRUD	Create, Read, Update, Delete
ITC	isothermal titration calorimetry
LE	Ligand Efficiency
NMR	Nuclear Magnetic Resonance
PDB	Protein Data Bank
ROC	Receiver Operating Characteristics
SILE	Size-Independent Ligand Efficiency
SPR	Surface Plasmon Resonance
