# Supplementary material for: DockNmine, a Web Portal to Assemble and Analyse Virtual and Experimental Interaction Data
Source: Int J Mol Sci. 2019 Oct 12;20(20):5062. doi: 10.3390/ijms20205062 (PMC6829441; doi:10.3390/ijms20205062)
Supplement: Supplementary file 1 [file ijms-20-05062-s001.pdf]

Supplementary materials

Receptor and ligand setup

The structure of GLUT1 was retrieved from the work of Deng an co-workers [1] avaibable under the Protein Data Bank (PDB) [2] under the id 4PYP. This structure was processed using autodockTools [3]: (i) water was removed, (ii) hydrogens were added, (iii) AD4 type atoms were assigned, (iv) Gasteiger charges were added to the structure. The resulting molecule was saved without any torsion definition in pdbqt format, heteroatoms were discarded. The bound ligand BNG ideal structure was downloaded from the PDB in order to keep the bound crystal structure for comparison. The sdf ideal file for the ligand was loaded in PyMol [4], saved into the mol2 format and then processed as follows using ADT: (i) input as a ligand, 26 non-polar hydrogens were added, torsion definitions were set to 14, (ii) the ligand was saved in pdbqt format. The same procedure was applied for all other ligands. The grid box was positioned at the center of the macromolecule, close to the center- or mass of the bound carbohydrate moiety. The docking parameters were kept to default values, and the vina config file was written. The time to process the BND ligand was 3 minutes on a Xeon E5-2620 using 24 cores, the energy of the best cluster for BND was -6.2 kcal/mol.

Sample for vina config file FOR GLUT1

```
receptor = 4pyp.pdbqt
center_x = 587.106
center_y = -31.483
center_z = 205.563
size_x = 18.75
size_y = 18.75
size_z = 18.75
```

Example of vina command-line call

```
# Adjust cpu number to your needs
vina --cpu 24 --config config.txt --exhaustiveness 100 --ligand BNG.pdbqt --out
BNG_out.pdbqt
```

Bibliography

1. Deng, D.; Xu, C.; Sun, P.; Wu, J.; Yan, C.; Hu, M.; Yan, N. Crystal structure of the human glucose transporter GLUT1. *Nature* **2014**, *510*, 121–125.
2. Burley, S.K.; Berman, H.M.; Bhikadiya, C.; Bi, C.; Chen, L.; Di Costanzo, L.; Christie, C.; Dalenberg, K.; Duarte, J.M.; Dutta, S.; et al. RCSB Protein Data Bank: biological macromolecular structures enabling research and education in fundamental biology, biomedicine, biotechnology and energy. *Nucleic Acids Res* **2019**, *47*, D464–D474.
3. Forli, S.; Huey, R.; Pique, M.E.; Sanner, M.F.; Goodsell, D.S.; Olson, A.J. Computational protein-ligand docking and virtual drug screening with the AutoDock suite. *Nat. Protocols* **2016**, *11*, 905–919.
4. Schrödinger, LLC The PyMOL Molecular Graphics System, Version 2.3 2015.
